# Developing genomic tools to assist turnip rape [*Brassica rapa* (L.) subsp.*oleifera* (DC.) Metzg.] breeding

**DOI:** 10.3389/fgene.2024.1435474

**Published:** 2024-08-28

**Authors:** Admas Alemu, Jagadeesh Sundaramoorthy, Kibrom B. Abreha, Muluken Enyew, Mulatu Geleta, Anders S. Carlsson

**Affiliations:** ^1^ Department of Plant Breeding, Swedish University of Agricultural Sciences, Alnarp, Sweden; ^2^ School of Biological Sciences, Washington State University, Pullman, WA, United States; ^3^ Institute of Biotechnology, Addis Ababa University, Addis Ababa, Ethiopia

**Keywords:** turnip rape, agro-morphological traits, genomic prediction, GWAS, QTL, SNPs

## Abstract

**Introduction:**

Turnip rape is recognized as an oilseed crop contributing to environmentally sustainable agriculture via integration into crop rotation systems. Despite its various advantages, the crop’s cultivation has declined globally due to a relatively low productivity, giving way to other crops. The use of genomic tools could enhance the breeding process and accelerate genetic gains. Therefore, the present research investigated 170 turnip rape accessions representing its global gene pool to identify SNP markers associated nine phenological and agro-morphological traits and estimate the genomic breeding values (GEBVs) of the germplasm through GWAS and genomic prediction analyses, respectively.

**Methods:**

Field trials were conducted at two sites in northern and southern Sweden to obtain the phenotypic data while genotyping was conducted via the genotyping-by-sequencing (GBS) method. The traits studied include days to flowering (DTF) and maturity (DTM), plant height (PH), seed yield (YLD), thousand seed weight (TSW), silique length (SL), number of siliques (NS), number of seeds per silique (SS), and pod shattering resistance (PSHR).

**Results and conclusion:**

Analysis of variance revealed substantial variation among accessions, with significant genotype-by-environment interaction for most traits. A total of 25, 17, 16, 14, 7, 5, 3, and 3 MTAs were identified for TSW, DTF, PH, PSHR, SL, YLD, SS and DTM, respectively. An 80%–20% training-test set genomic prediction analysis was conducted using the ridge regression – BLUP (RR-BLUP) model. The accuracy of genomic prediction for most traits was high, indicating that these tools may assist turnip rape breeders in accelerating genetic gains. The study highlights the potential of genomic tools to significantly advance breeding programs for turnip rape by identifying pivotal SNP markers and effectively estimating genomic breeding values. Future breeding perspectives should focus on leveraging these genomic insights to enhance agronomic traits and productivity, thereby reinstating turnip rape as a competitive and sustainable crop in Sweden and broader global agriculture.

## Introduction

*Brassica rapa* L. (syn. *B. campestris*) (AA, 2n = 20) is the most widely distributed and cultivated member of the genus *Brassica* within the family *Brassicaceae* (Gómez-Campo and Prakash, 1999; Kaur et al., 2021a; Wu et al., 2022). It is one of the earliest domesticated *Brassica* species and wild progenitors of the allotetraploids *Brassica juncea* (AABB, 2n = 36) and *Brassica napus* (AACC, 2n = 38) (Gómez-Campo and Prakash, 1999; Chalhoub et al., 2014; Wu et al., 2022). It comprises several subspecies and morphotypes cultivated as leafy vegetables (Broccoletto – *B*. *rapa* ssp. *oleifera*; Chinese cabbage – ssp. *pekinensis* and Buk choy – ssp. *chinensis*); root vegetable (Turnips – *B. rapa* ssp. *rapa*); oilseed crops (turnip rape – *B. rapa* ssp. *oleifera* and brown sarson – ssp. *dichotoma)* and fodder crops (Fodder turnip – *B. rapa* ssp. *rapifera*) (Gómez-Campo and Prakash, 1999; Shyam et al., 2012; Cartea et al., 2021; Wu et al., 2022). Genomic inferences and anecdotal pieces of evidence suggest the origin of *B. rapa* as European-Central Asian (Qi et al., 2017; Kaur et al., 2021a). Previous genetic and phylogenetic analyses grouped *B. rapa* into five subgroups reflecting the morphology and geographical distributions. These are i) Asian turnip and oilseed cultivars; ii) European turnip and oilseed cultivars; iii) yellow and brown sarson; iv) Chinese cabbage; and v) Pakchoi, choi sum and tatsoi (Bird et al., 2017; Vélez-Gavilán, 2018). Factors including biased gene retention, genetic redundancy and functional diversification are believed to be the major contributors to the widespread of the various morphotypes and subspecies (Lysak et al., 2005; Tang and Lyons, 2012; Kaur et al., 2021a).

*Brassica rapa* has been cultivated in several parts of the world including Asia and Europe, creating genetically highly diverse subspecies, morphotypes and cultivars. For instance, some leafy vegetables, such as Chinese cabbage (ssp. *pekinensis*), have believed to be evolved naturally in Central China from Pak choi (ssp. *chinensis*) and turnip rape (ssp. *oleifera*) followed by artificial selection for adaptation to different environments and different purposes (Lee, 1982). Around the 14th century, the huge demand increment for vegetable oil led to the introduction of *turnip rape B. rapa* ssp. *oleifera* in temperate regions of European countries (Reiner et al., 1995; Guo et al., 2014).

Turnip rape (*B. rapa* ssp. o*leifera*) is the third most important Brassica oilseed crop next to *B. napus* and *B. juncea* widely grown in China, Canada, India, and northern Europe Wu et al., 2022. It has been identified as a rich source of human nutrition as it contains up to 46% oil, 38% proteins and more than 20% carbohydrates in the seeds on a dry weight basis (Bassegio and Zanotto, 2020; Kaur et al., 2021a; Wanasundara et al., 2024). However, turnip rape is currently cultivated in a very small area globally compared to its allotetraploid relatives *B. napus* and *B. juncea* (Kaur et al., 2021a).

Forage and grain fodder crops have been predominantly incorporated into Northern European agriculture via crop rotation. They have shown several benefits by contributing to sustainable and environment-friendly agriculture. Spring turnip rape is a well-adapted and environmentally sustainable oil crop in crop rotations. Spring turnip rape cultivars are widely known because they mature earlier than oilseed rape cultivars making them ideal for temperate countries in the northern hemisphere. In Sweden, turnip rape has been identified as an environmentally sustainable oilseed crop with the potential to be incorporated into crop rotation systems, primarily in northern Sweden, but it is also a viable alternative for cultivation in the Svealand (Central Sweden) parts of (https://www.slu.se/en/Collaborative-Centres-and-Projects/grogrund/projekt/rybs---en-flexibel-och-talig-oljegroda-for-sverige/).

Turnip rape was one of the largest oil crops in terms of area coverage in Sweden for a few years in the 1980s, predominantly grown in the temperate latitudes of the northern hemisphere from 55°N to 65°N of the country. However, in the late 1990s, spring turnip rape cultivation declined, mainly due to the lack of investment in improvement programs leading to the termination of spring turnip rape breeding programs in the country (Persson, 2017). This led to a lack of high-yielding improved varieties with the ability to withstand recurring biotic and abiotic stresses, resulting in a significant reduction in the crop’s cultivation area, with only 13,000 ha at present (Jordbruksverket, 2022).

Expanded accessibility along with the cost-effectiveness of high throughput genomic data of various crops revolutionized plant breeding shifting from phenotypic to genomic-based selection. Shortly after the genotyping revolution (starting in the 1980s), a large number of linkage and association mapping studies were conducted in various crops to identify quantitative trait loci (QTL) controlling different traits of interest (Bernardo, 2008). Genome-wide association studies (GWAS) have been extensively used in several Brassicaceae crops including *B. rapa* to identify QTLs or quantitative trait nucleotides (QTNs) using a collection of different historical germplasms of a particular crop species with highly accumulated historical recombination events (Yano et al., 2016; Challa and Neelapu, 2018; Alemu et al., 2021b; Tibbs Cortes et al., 2021; Alemu et al., 2022). The complex nature of QTLs identified via GWAS from diverse germplasm sets makes the approach challenging for direct and immediate genomic-enabled breeding applications. Genome-wide prediction/selection has emerged as a powerful genomic-assisted breeding method by alleviating the limitations of methods that utilize GWAS and bi-parental QTL mapping-derived markers. Genomic prediction (GP) estimates individual genotypes’ breeding values based on overall genetic merits. Hence, GP predicts breeding values [also known as genomic estimated breeding values (GEBVs)] of candidate genotypes instead of identifying QTLs linked to a particular trait. Compared to conventional phenotypic-based selection, genomic prediction-based selection reduces both the cost and required time per breeding cycle and enhances testing efficiency in field evaluations conducted to develop a crop variety in several breeding programs (Alemu et al., 2024).

The objectives of this study were to identify SNP markers associated with nine phenological and agro-morphological traits of turnip rape via GWAS and estimate the breeding values of turnip rape collections in terms of these traits via genomic prediction. To accomplish this 170 turnip rape accessions representing a worldwide collection were assembled and characterized for the nine traits by growing them at two field experimental sites. Genotyping-by-sequencing (GBS) method was used for genotyping accessions to generate SNP markers.

## Materials and methods

### Plant material

The germplasm panel used in this study comprises 170 turnip rape accessions that include cultivars, breeding populations, landraces, and wild populations originated from at least 26 countries across the globe. Fifty of the 170 accessions were of Swedish origin while the vast majority of the other accessions originated from Italy (22), India (17), Canada (12), Germany (12), China (10), Finland (8), Bangladesh (7), United States (4), Georgia (3), Pakistan (3), Austria (2), Colombia (2), Denmark (2) and Slovakia (2). Australia, Cuba, Iran, Nepal, Poland, Portugal, the Russian Federation, Spain, Tibet, Turkey and United Kingdom were represented by a single accession each. The countries of origin of the remaining three accessions are unknown (Supplementary Table S1). These accessions were procured from several gene banks including the Leibniz Institute of Plant Genetics and Crop Plant Research (IPK – 73 accessions), Plant Gene Resources of Canada (PGRC – 36 accessions), Nordic Genetic Resource Center (NordGen – 33 accessions), Centre for Genetic Resources, the Netherlands (CGN – 8 accessions), the National Plant Germplasm System, United States (NPGS – 5 accessions) and A&A Seed Farms, Canada (A&A Canada – 2 accessions). In addition, Jerrestad Agro AB, Sweden (a turnip rape breeding company) provided 13 turnip rape cultivars (Supplementary Table S1).

### Experimental design, phenotypic data collection and phenotypic plasticity analysis

Field trials were conducted in 2021 using 170 accessions at two locations in Sweden: SITES, Lönnstorp (5539′N, 1306′E) and Öjebyn Agro Park (6521′N, 2124′E), representing different agro-ecological zones. The field experiment was laid out in an alpha lattice design comprising three replications and 17 blocks with 10 plots each. The plot size was 3 m^2^ (3 m × 1 m) and seeds were planted in six rows in each plot. Subsequently, thinning was done at the seedling stage to maintain a spacing of 10 cm between plants in each plot. In total, phenotypic data of nine different phenotypic traits of turnip rape were collected from each plot of one or both experimental sites. The data for days to flowering (DTF), plant height (PH), number of siliques (NS), silique length (SL), pod shattering resistance (PSHR), and thousand seed weight (TSW) were collected from both (Öjebyn and Lönnstorp) experimental sites. The phenotypic data for days to maturity (DTM) and seed yield per plot (YLD) were collected only from Lönnstorp while the data for seeds per silique (SS) was collected only from Öjebyn. DTF was recorded for each plot as the number of days taken from sowing to the flowering of half of the plants on the plot. DTM was measured as the number of days that have elapsed from sowing to physiological maturity of plants on the plot as indicated by the yellowing of the siliques. PH was measured as the distance from the base of the aboveground plant to the tip of the main inflorescence at maturity. The PSHR was visually evaluated and recorded as low, intermediate and high at maturity. A total of 1,000 seeds/plot was cleaned, counted with a seed counter and TSW determined. The number of siliques per plant (NS) on each plot was determined based on five representative plants from the middle of the plot at maturity. The number of seeds/silique (SS) and silique length (SL) were counted and measured, respectively, based on 10 representative siliques sampled from the middle part of each plot. Seed yield per plot (YLD) was measured after machine-harvesting the whole plot and cleaning.

Phenotypic data analysis from single and combined environment were evaluated using the Multi-environment Trial Analysis with R (META-R) (Alvarado et al., 2020). The genotypic and environmental variances and adjusted phenotypic mean values [best linear unbiased predictions (BLUPs)] of the studied traits was estimated including the genotypes, locations, blocks and replications. The variances of a single environment and BLUPs were estimated using a linear model:

$$Y_{ijk}=u+R_{i}+B_{j}\left(R_{i}\right)+G_{k}+\varepsilon_{ijk}$$

where $Y_{ijk}$ is the target trait phenotypic value of the $K_{i}th$ genotype in the $R_{i}th$ replication and $B_{i}th$ block; u is the intercept; $R_{i}$ is the i th replicate effect; $B_{j}\left(R_{i}\right)$ is the effect of j th block nested in i th replicate; $G_{k}$ is k th genotype effect; and $\varepsilon_{ijk}$ is the error associated with the i th replicate, j th block and k th genotype with the assumption of independent and identically distributed normal random variables (iid) with mean zero and variance $\delta^{2}\varepsilon$ .

For the combined environment, ANOVA and BLUP analyses were conducted as follows:

$$Y_{ijkl}=u+E_{i}+R_{j}\left(E_{i}\right)+B_{k}\left(E_{i}R_{i}\right)+G_{l}+E_{i}\times G_{l}+\varepsilon_{ijkl}$$

where $E_{i}$ is the $E_{i}$ th environment effect and $E_{i}\times G_{l}$ is the effect of genotype-by-environment interaction (G×E).

Trait repeatability (*H*) was analyzed using the phenotypic values scored across replicates within a location as:

$$H=\frac{\delta^{2}g}{\delta^{2}g+\delta^{2}E/nReps\;}$$

where $\delta^{2}g$ is the genotypic variance; $\delta^{2}E$ is the estimated error and nReps is the number of replications.

The broad-sense heritability (*H* ^*2*^) of the traits was calculated as:

$$H^{²}=\frac{\delta^{2}g}{\delta^{2}g+\delta^{2}ge/nEnvs+\delta^{2}E/\left(nEnvs\times nReps\right)\;}$$

where $\delta^{2}ge$ is the G×E variance; nEnvs is the number of environments.

The Pearson correlation coefficient analysis conducted in the *metan* package in R (Olivoto et al., 2020).

The Bayesian Finlay-Wilkinson regression model (FWR) was employed to estimate accessions phenotypic plasticity following the two stage ordinary least squares (OLS) method in the *FW* package (Lian and de Los Campos, 2016) with a model:

$$Y_{ij}=\mu +g_{i}+\left(\;b_{i}+1\right)\;h_{i}+\varepsilon_{ij}$$

where $Y_{ij}$ is the phenotype of the ith accession measured in the jth environment, μ represents the intercept as the population mean, $g_{i}$ is the main genetic effect of the ith accession, $h_{i}$ is the environmental effect of the jth environment, $b_{i}+1$ indicates the change in performance of the ith accession per unit change in the environmental effect ( $h_{i}$ ), and $\varepsilon_{ij}$ is the residual error. Hence, FWR decomposes the phenotype of individual accessions into three components: genotypic main effect, a regression slope, and residual value. The regression slope, also called linear plasticity, measures the linear response of a genotype to environmental changes relative to other genotypes in the population. The linear plasticity score of accessions were used to identify SNP markers and genomic regions controlling phenotypic plasticity of the six traits measured in both environments.

### DNA extraction, sequencing, read alignment and variant discovery

The seeds of 165 accessions were planted in a greenhouse at the Department of Plant Breeding, Swedish University of Agricultural Sciences (SLU), Sweden. Fifty-cell (5 × 10) plastic seedling trays filled with soil were used for planting. For each accession, leaf tissue was sampled from 100 two-week-old seedlings and pooled into a single Eppendorf tube for DNA extraction. Each seedling was represented by a 6 mm disc sampled using a Unicore Punch Kit (Qiagen). Genomic DNA was extracted from each pooled sample of each accession using the Thermo Scientific GeneJET Genomic DNA Purification Kit. Following extraction and purification, a high-quality genomic DNA sample of each accession was transferred to a single well of a 96-well plate. Subsequently, sealed 96-well plates containing the samples were dispatched to LGC Genomics GmbH (Berlin, Germany) for genotyping.

SNP genotyping was employed using the genotyping-by-sequencing (GBS) method at the LGC Genomics GmbH following the method by Elshire et al. (2011) with minor modifications. Briefly, pooled DNA samples of individual accessions were digested with MsII (5′-CAYNN*NNRTG-3′, a 5 bp cutter) restriction enzyme based on the recommendation of the experts of the LGC, Biosearch Technologies. An average insert size of 215 bp was produced by the restriction enzyme, suitable for sequencing on Illumina platforms. The restriction fragments were ligated to unique sequence barcode adapters. Equal aliquots of adapter-ligated DNA samples were pooled in a single tube to produce sequencing libraries. The pooled DNA was amplified with the sequencing primer and purified using a QIAGEN PCR purification kit. The purified DNA was quantified and sequenced with 2 × 150 bp mode on the Illumina NextSeq 500/550 v2 and NovaSeq FC NGS platforms.

The in-house Illumina bcl2fastq v2 software package was used for demultiplexing of samples based on the barcode adapters ligated to individual DNA samples followed by verification of restriction site. This was followed by adapter-clipping, discarding of reads containing Ns and whose 5′-ends did not match the restriction enzyme site, trimming the 3′-end of the remaining reads to obtain an average Phred quality score of ≥20 over a window of ten bases, and discarding reads with a final length <20 bp. *B. rapa* reference genome assembly CAAS_Brap_v3.01 (https://www.ncbi.nlm.nih.gov/datasets/genome/GCF_000309985.2/; 352.8 Mb) was used as a reference for sequence analysis. The quality trimmed reads were aligned against the reference genome using the BWA-MEM software package version 0.7.12 (Li, 2013). A 95.3% mapping rate was obtained between the GBS reads and the reference genome. Then, Freebayes v1.0.2-16 (Garrison and Marth, 2012) was used for variant discovery and allele count. The alignment of the reads of the 165 turnip rape accessions resulted in the discovery of 653,362 SNPs. Variants were filtered using a GBS-specific rule set. The total number of fully covered SNPs in 91% of the samples with a minimum read count of 100 was 24,856. Further filtering of these SNPs to obtain bi-allelic SNPs with no missing data, 5% minor allele frequency (MAF), and a maximum of 20% samples having both alleles resulted in 2,327 SNPs, which were used for marker-trait association analysis and genomic prediction.

Then, barcode sequences were removed clipped from the read sequences followed by trimming of the reads using the in-house software Trimmomatic-0.33. The SNP polymorphism discovery of filtered reads was made by mapping to the *B. rapa* reference genome assembly (https://www.ncbi.nlm.nih.gov/datasets/genome/GCF_000309985.2/). The filtering of variants and the minimum read depth was applied according to the GBS-specific ruleset.

### Genome-wide association study and linkage disequilibrium analysis

**FIGURE 1:**
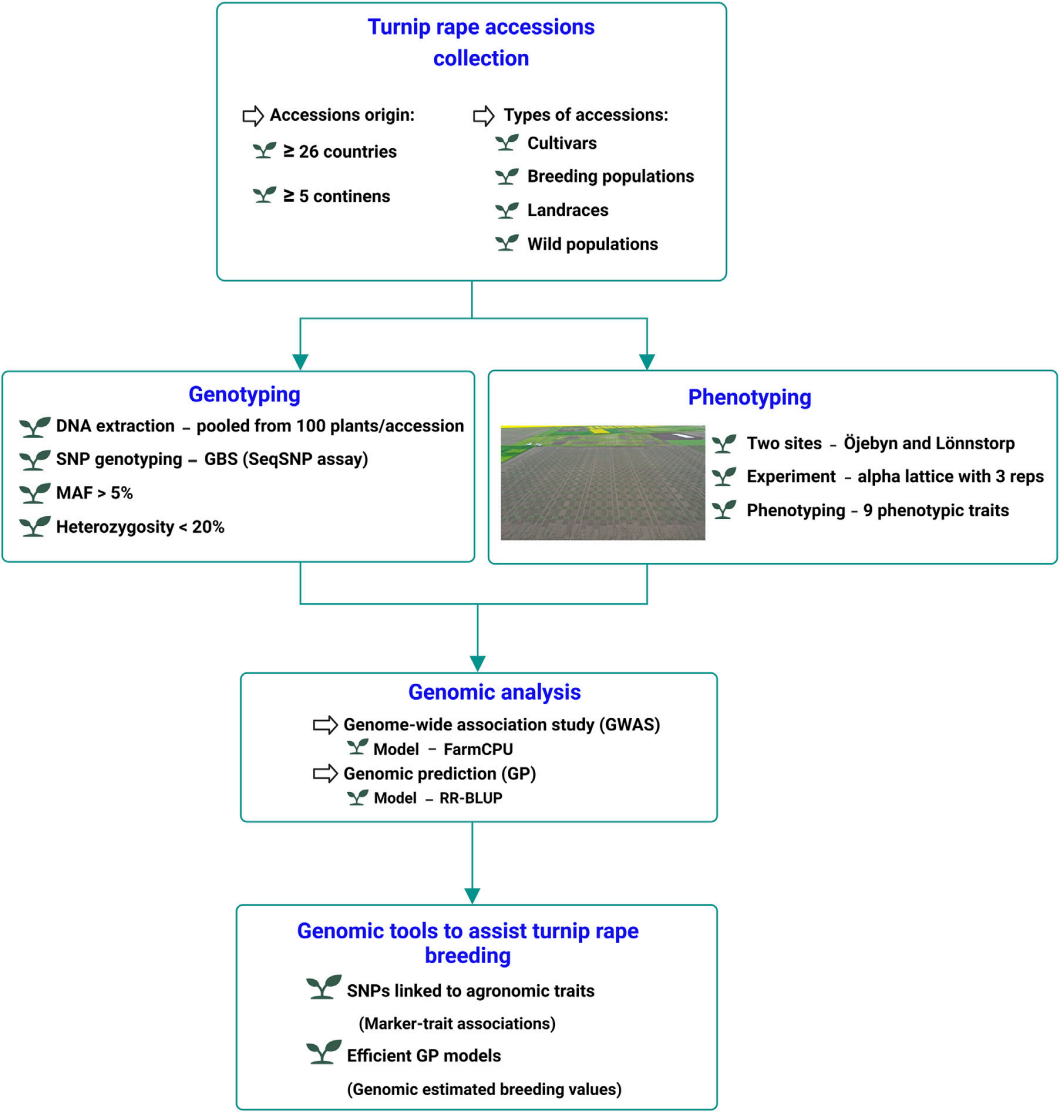
Schematic presentation of the experimental setup for the GWAS and genomic prediction analysis of nine phenotypic traits of turnip rape.

The overall design of the current research is illustrated in Figure 1. Adjusted phenotypic mean values (BLUPs) of the studied traits collected from the 170 turnip rape accessions along with their SNP marker profile were employed for GWAS analysis. The analysis was conducted using Fixed and random model Circulating Probability Unification (FarmCPU) (Liu X. et al., 2016). The model was implemented within the package GAPIT v.3 (Wang and Zhang, 2021) in R environment v.4.3.2 (R Core Team, 2023) using the default “method.bin” parameter as “optimum.” FarmCPU is one of the most robust GWAS models, controlling confounding factors and false positive marker-trait associations (Liu X. et al., 2016). The model applies a genomic kinship matrix (K) developed from pseudo-quantitative trait nucleotides (QTNs) as random effects. The default “method.bin” parameter in FarmCPU applies two functions: i) *bin.size* = c (5e5, 5e6, 5e7) used for segmentation of the whole genome into bins with particular kilo base pairs representing the window size to select possible quantitative trait nucleotides (QTNs); and ii) *bin.selection* = seq (10, 100, 10) to specify the number of QTNs that could be selected and used in the FarmCPU model as covariates in each loop (Liu X. et al., 2016). The model implements two iterative steps. The first step is the fixed effect model (FEM) while the second step is the random effect model (REM) (Liu X. et al., 2016). In the current GWAS study, the model can be interpreted as follows:

Step 1 (FEM):

$$y_{a}=M_{a1}Q_{1}+M_{a2}Q_{2}+\ldots +M_{an}\;Q_{n}+M_{a1}\;Q_{1}+S_{at}\;e_{t}+\varepsilon_{a}$$

Step 2 (REM):

$$y_{a}=J_{a}+\varepsilon_{a}$$

where $y_{a}$ is the BLUPs of the target trait’s phenotypic values recorded for the a ^th^ accession and $\varepsilon_{a}$ is the residual. In the first step (FEM), $M_{a1},M_{a2},\ldots ,M_{an}$ are genotypes of n pseudo-QTNs; $Q_{1},Q_{2},\ldots ,Q_{n}$ represent the corresponding effects from pseudo-QTNs; $S_{at}$ is a genotype score of a th accession at the t th SNP marker and $e_{t}$ is the corresponding effect of the t th SNP marker. In the second step (REM), $J_{a}$ is the total genetic effect of the a th accession derived from the variance-covariance matrix as $G=2K\delta_{a}^{2}$ , where K is the kinship derived from the pseudo-QTNs and $\delta_{a}^{2}$ is SNPs genetic variance estimated in the REM step of the model (Liu X. et al., 2016).

The exploratory and Bonferroni-corrected thresholds were applied to identify SNP markers significantly associated with the studied traits. Here, the most commonly applied exploratory threshold with a *P*-value ≤0.0001 or [ ${-\log}_{10}\left(P-value\right)\geq 4$ ] was applied to identify nominal marker-trait associations. In addition, the Bonferroni threshold adjusted for multiple marker tests at *P* ≤ 0.05 was applied to exclude potential false positive marker-trait associations. It was estimated as ${\text{Bonferroni}\;\text{threshold}=-\log}_{10}\left(\;\frac{t}{n}\;\right)$ where t is the overall false positive threshold (0.05) and n is the number of SNP markers used in the current GWAS analysis (2,328). Therefore, the Bonferroni threshold was calculated as ${-\log}_{10}\left(\frac{0.05}{2328}\right)=4.67$ .

The pairwise linkage disequilibrium (LD) between SNP markers was calculated using *r* ^2^ values in TASSEL (Bradbury et al., 2007) in a full matrix size.

The GWAS analysis for phenotypic plasticity was conducted for DTF, TSW, PSHR, NS, PH and SL measured across the two environments.

### Genomic prediction and cross-validation analysis

The potential of genomic prediction to assist turnip rape breeding was evaluated using one of the most efficient and widely applied ridge-regression BLUP (RR-BLUP) using the *rrBLUP* package in R (Endelman, 2011). The RR-BLUP model was implemented using the following mixed linear model:

Y=μ+Zβ+ε

where Y is the *N* × 1 vector values of the BLUPs recorded for a studied trait; μ is a vector of trait’s overall phenotypic mean; and Z is the *N* × *N*_*m*_ SNP marker matrix. *N* and *N*_*m*_ are the number of accessions and SNP markers, respectively, applied to the current genomic prediction analysis. β is the *N*_*m*_ × 1 vector of random SNP effects calculated using the “*mixed.solve*” function in the RR-BLUP package following $\beta \;∼\;N\left(0,I\delta_{g}^{2}\right)$ , where I is the identity matrix and $\delta_{g}^{2}$ is the genetic variance component contributed by SNP markers; and ε is the *N* × 1 is the vector of residuals.

The accuracy of the prediction models was assessed through a cross-validation method using 80% of the accessions as a training set (TRS) and the remaining 20% as a test set (TS). Here, the genomic estimated breeding values (GEBVs) of accessions in the TS were estimated with the genomic prediction models trained on the TRS accessions. Then, Pearson’s correlation coefficient between the GEBVs and adjusted phenotypic values (BLUPs) was recorded as genomic prediction ability, which was repeated 500 times for each trait. The prediction accuracy was estimated by dividing the prediction ability by the square root of the trait’s broad-sense heritability (Legarra et al., 2008; Chen et al., 2011; Alemu et al., 2021a).

## Results

### Analysis of phenotypic variance, plasticity, correlation and heritability

Analysis of variance (ANOVA) for phenotypic data was conducted within and across sites (environments). At the Lönnstorp site, genotypic variance was highly significant (*p* < 0.0001) for all traits except NS and SL (Supplementary Table S2). Thousand seed weight (TSW) had the highest repeatability (0.95) followed by PH, DTF and YLD, which had repeatability values of 0.89, 0.82, and 0.7, respectively. However, SL and NS had low repeatability values of 0.21 and 0.22, respectively. At the Öjebyn site, the genotypic variance of all seven studied traits was highly significant. TSW and DTF had the highest repeatability score (0.94) while SS exhibited the lowest repeatability value (0.47) (Supplementary Table S2).

The combined environment ANOVA was conducted for six of the nine traits with data from the two sites. In this case, DTF, PH, SL and TSW exhibited highly significant variation between accessions (Table 1). The variance due to G×E was highly significant for DTF, NS, PSHR, and TSW. There was no significant G × E in PH and SL. Broad-sense heritability (H^2^) was high for PH, DTF and TSW with values of 0.85, 0.80, and 0.73, respectively. Other traits had low to moderate broad-sense heritability (Table 1).

**TABLE 1:** Analysis of variance and broad-sense heritability of six phenotypic traits of turnip rape conducted based on data collected from both Öjebyn and Lönnstorp field trials.

Statistics	DTF	PH	NS	SL	PSHR	TSW
σ^2^G	2.33	65.51	26	0.08	0.04	0.11
σ^2^GxE	0.90	0.92	82.68	0.00	0.07	0.07
σ^2^E	0.86	66.68	334.75	0.41	0.29	0.03
Mean	35.76	81.34	58.55	5.95	2.48	2.76
CV	2.59	10.04	31.25	10.71	21.84	6.61
H^2^	0.80	0.85	0.21	0.55	0.32	0.73
Gen. Sign	***	***	NS	***	*	***
GxE sign	***	NS	***	NS	***	***

DTF, days to flowering; PH, plant height; NS, number of siliques; SL, silique length; PSHR, pod shattering resistance; TSW, thousand seed weight.

**FIGURE 2:**
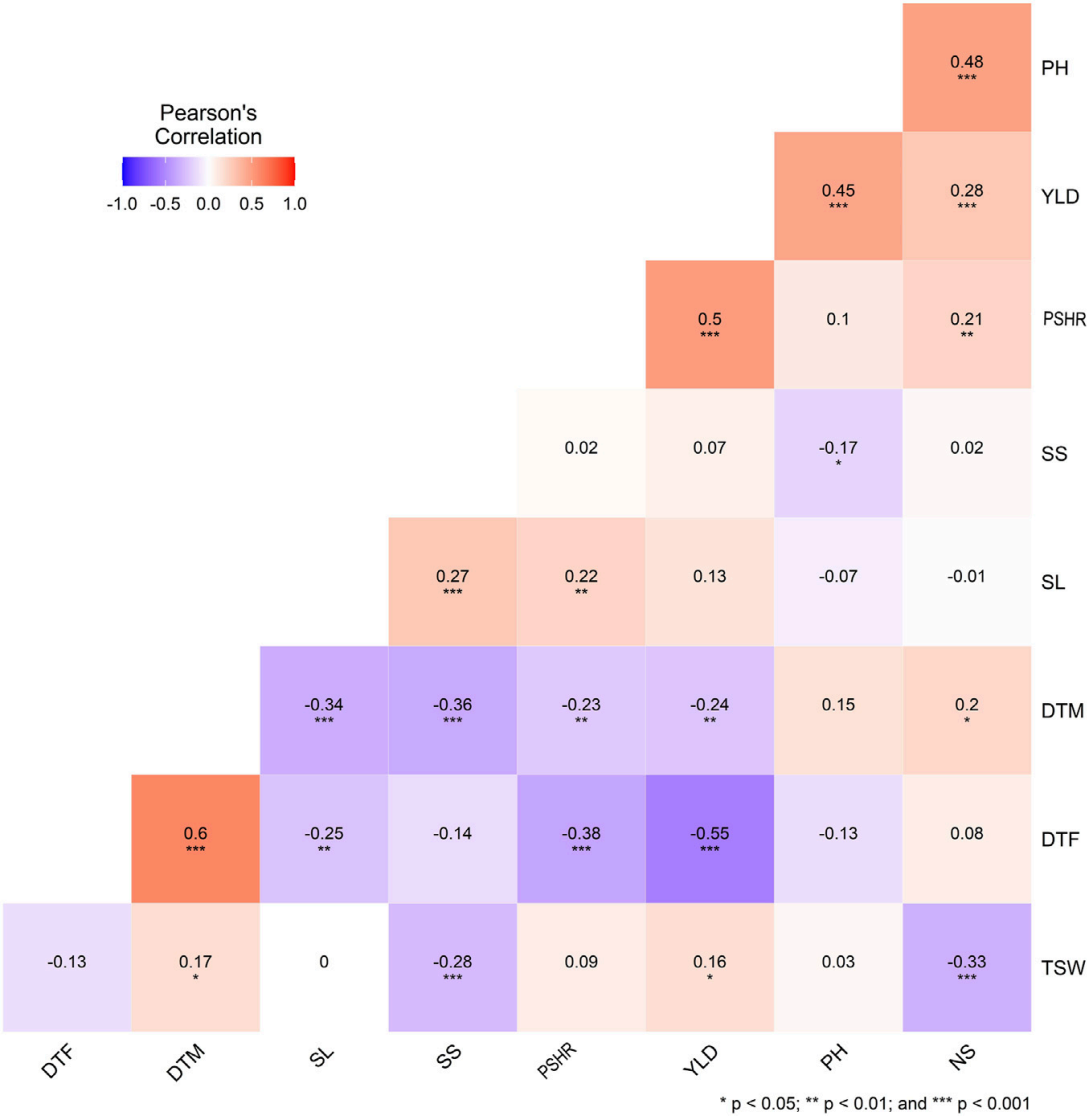
Correlation coefficients and significance levels for agronomic traits in 170 turnip rape accessions. DTF, days to flowering; DTM, days to maturity; SL, silique length; SS, number of seeds per silique; PSHR, pod shattering resistance; YLD, yield per plot; PH, plant height; NS, number of silique; TSW, thousand seed weight.

Strong correlations, both positive and negative, were evident among the traits examined, as illustrated in Figure 2. The number of days to flowering (DTF) was significantly negatively correlated with yield (*r* = −0.55, *p* < 0.001), PSHR (*r* = −0.38, *p* < 0.001) and SL (*r* = −0.25, *p* < 0.01) and positive correlation with DTM (*r* = 0.60, *p* < 0.001). Seed yield (YLD) demonstrated significant positive correlations with PSHR (r = 0.50, *p* < 0.001), PH (r = 0.45, *p* < 0.001), NS (r = 0.28, *p* < 0.001), and TSW (r = 0.16, *p* < 0.05). Moreover, TSW exhibited a significant negative correlation with both NS and seed SS (Figure 2).

**FIGURE 3:**
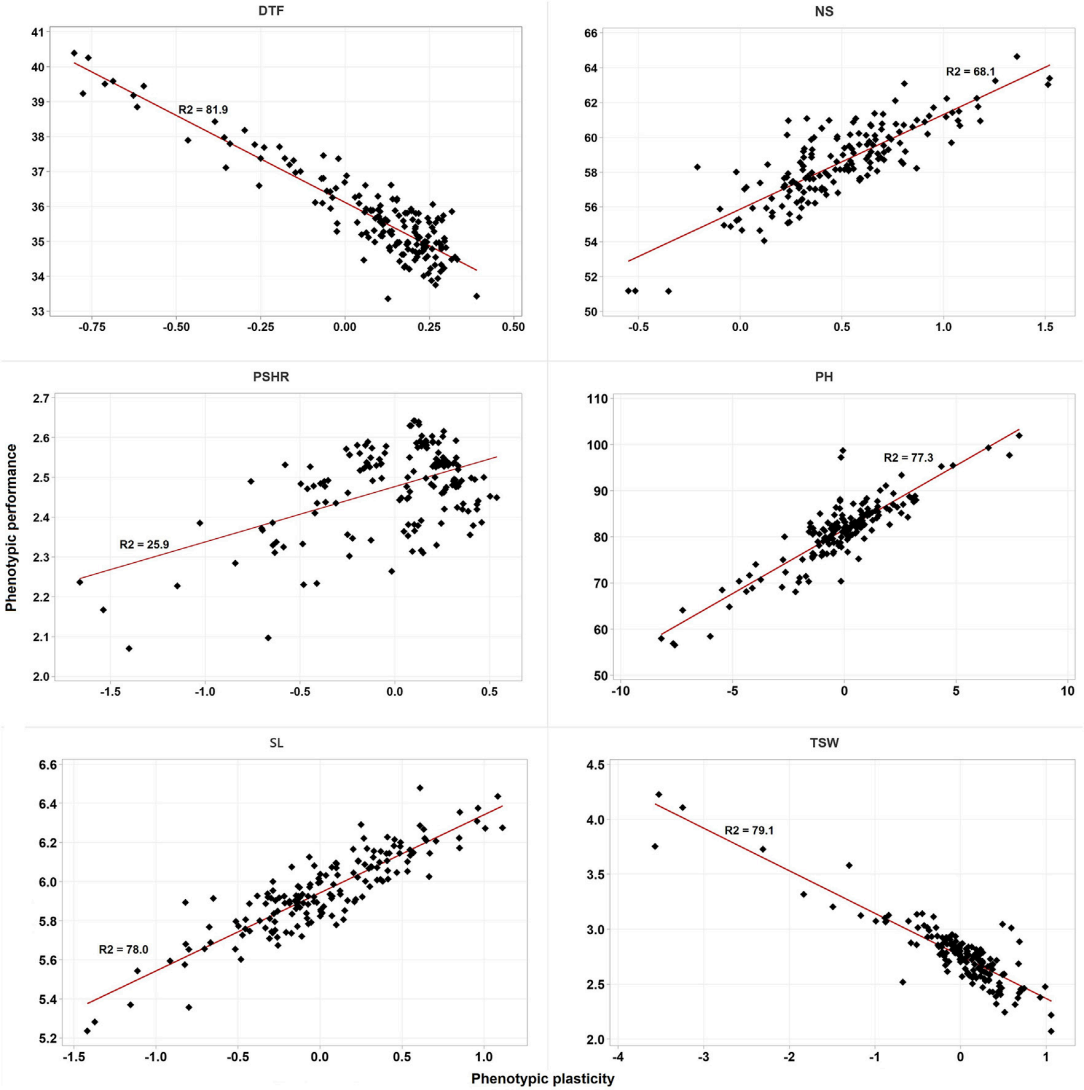
Scatter plot of the BLUPs estimated phenotypic performance of accessions against their estimated phenotypic plasticity. DTF, days to flowering; NS, number of siliques; PH, plant height; PSHR, pod shattering resistance; SL, silique length; TSW, thousand seed weight. R2 is the coefficient of determination.

Except for PSHR, the estimated phenotypic plasticity of the five traits showed a strong correlation (both positive and negative) with the estimated BLUP values for the combined phenotypic performance of accessions across the two environments. Specifically, DTF and TSW exhibited a negative correlation with phenotypic plasticity, whereas NS, SL, and PH demonstrated positive correlations (Figure 3).

### Linkage disequilibrium and marker-trait associations identified via GWAS

**FIGURE 4:**
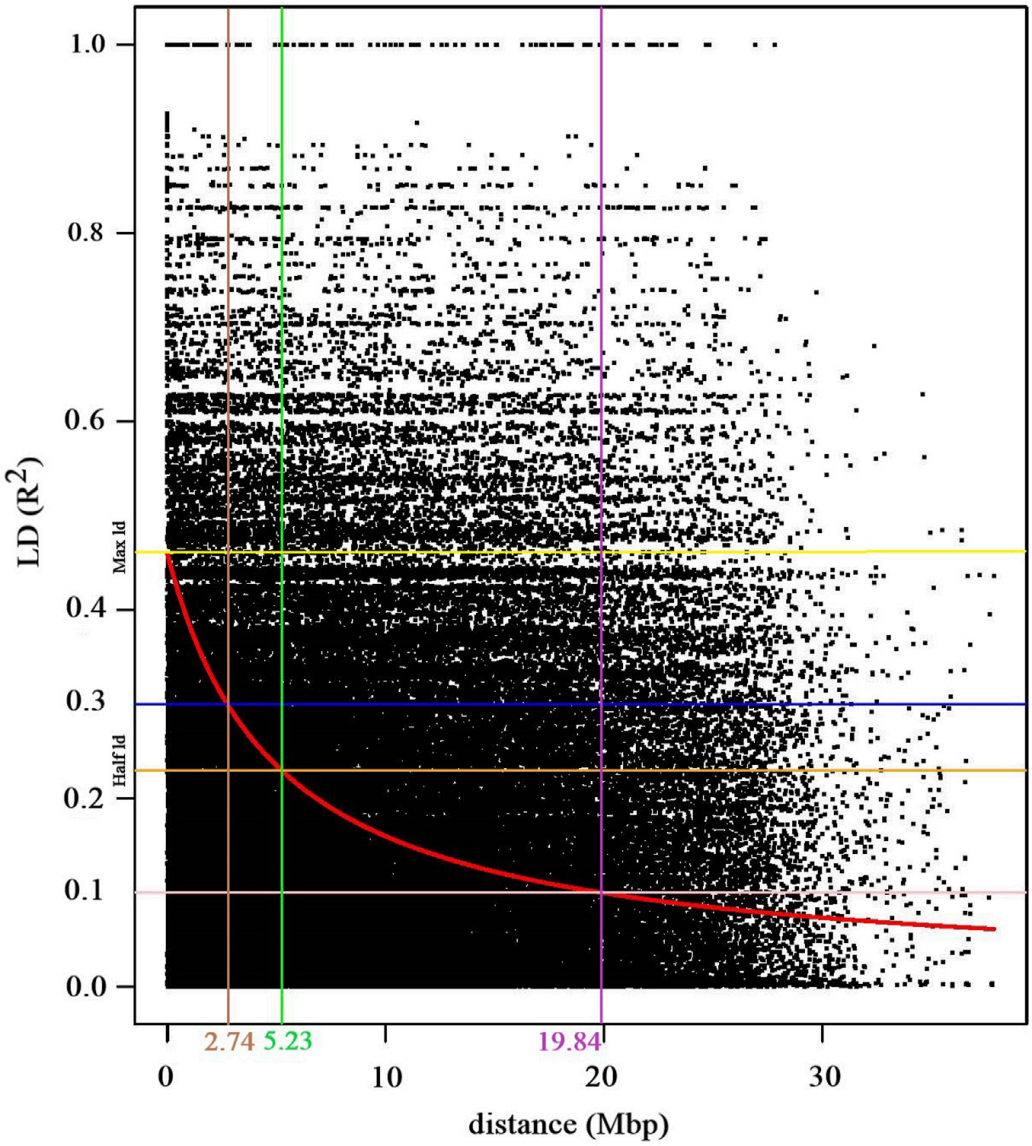
Scatter plot depicting the genome-wide linkage disequilibrium (LD) decay curve, which plots pairwise SNPs *r* ^2^ values against their physical distances in mega base pairs (Mbp). The red curve line indicates the LD decay trend, which was derived by fitting the smoothing spline regression model (loess). The yellow and orange horizontal lines indicate the pairwise *r* ^2^ values at the maximum and half LD decay, respectively. The blue (*r* ^2^, 0.3) and pink (*r* ^2^, 0.1) horizontal lines intersects with the LD decay cure at 2.74 Mbp (brown vertical line) and 5.23 Mbp (pink vertical line), respectively. The green vertical line indicates the distance (5.23 Mbp) where the LD decay curve intersects with the half LD decay.

A total of 2,706,301 pairwise linkage disequilibrium (LD) comparisons were derived from the full matrix analysis with the currently applied SNP markers. The genome-wide linkage disequilibrium (LD) decay analysis demonstrated a rapid decay of LD. Initially, the maximum LD started at an *r* ^2^ value of 0.44 and reached to half LD decay at *r* ^2^ = 0.23 (Figure 4). Notably, the LD decay was rapid as the *r* ^2^ value decreased from 0.3 to 0.1, the corresponding genomic distance increased substantially from 2.74 to 19.84 megabase pairs (Mbp) (Figure 4).

**FIGURE 5:**
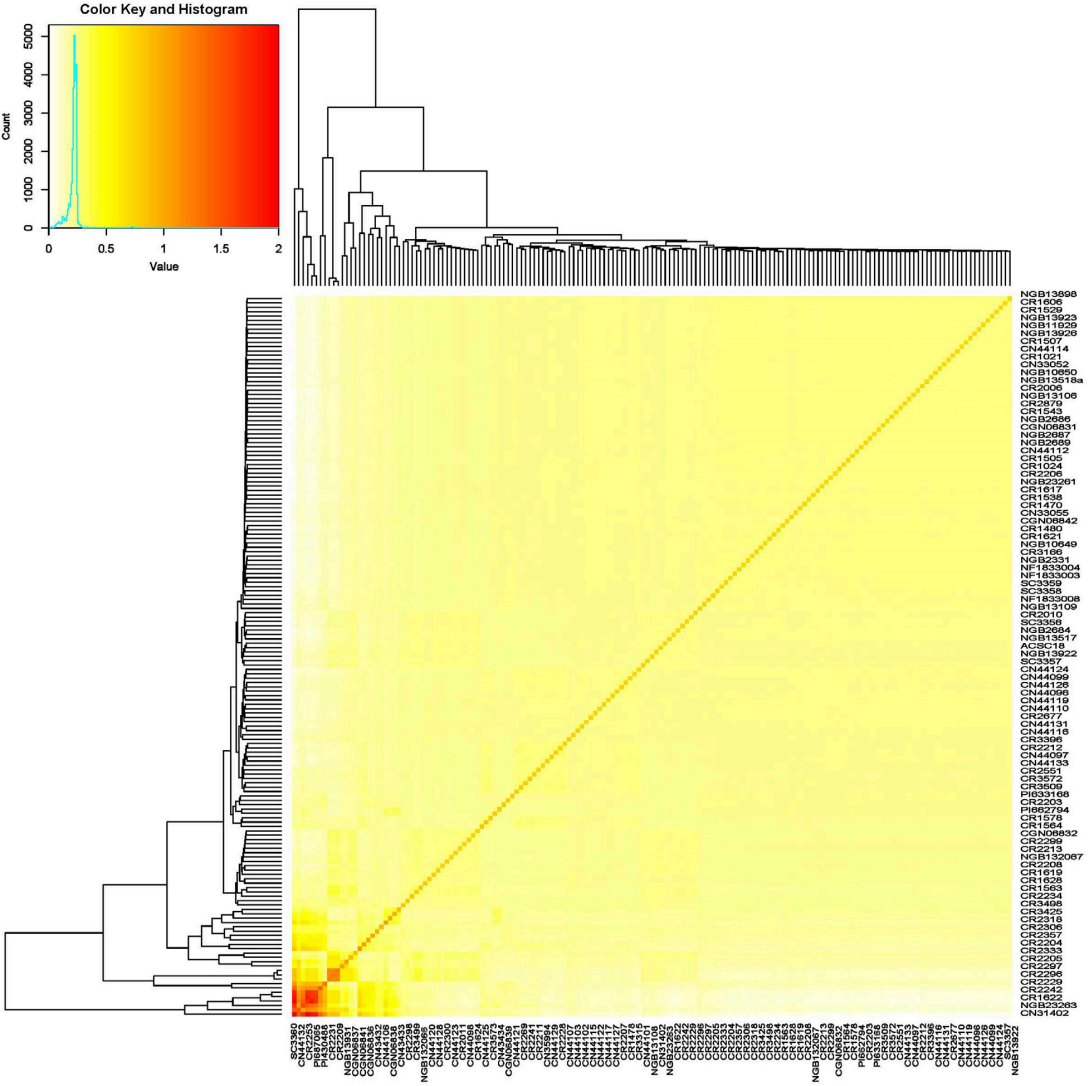
Heat map of the turnip rape panel comprising accessions representing its global gene pool based on their SNP marker profile revealing the absence of a strong population structure.

The germplasm panel used in this study comprised accessions from more than 27 countries across five continents (Supplementary Table S1). However, the SNP marker-based kinship and population structure analysis revealed poor population structure, as the vast majority of the accessions were clustered together and there was no clear clustering of accessions based on factors such as country or region of origin (Figure 5).

**FIGURE 6:**
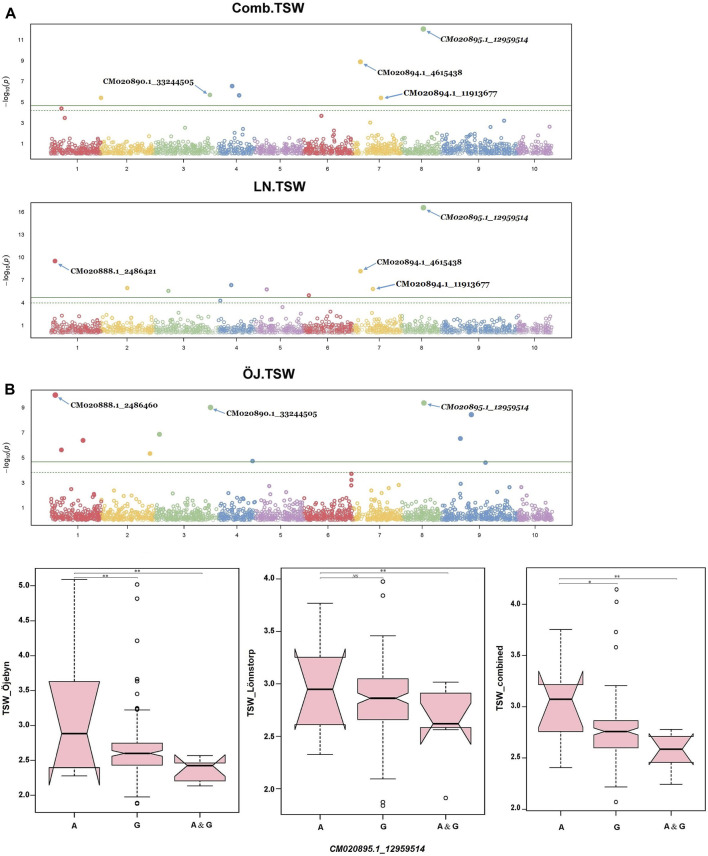
Manhattan plot **(A)** depicting significant SNP markers across the ten chromosomes associated with thousand seed weight (TSW) identified for the Öjebyn site, Lönnstorp site and combined environment, and box plots **(B)** depicting significant variation in TSW between groups of turnip rape accessions grouped based on whether they have only allele A or allele G or both at the SNP marker *CM020895.1_12959514*, for the two sites and combined environment. *p < 0.05; **p < 0.01; NS, non-significant.

Several significant marker-trait associations (MTAs) were identified via GWAS analysis for the nine studied traits. In total, 65 SNP markers exhibited significant associations with one or more traits for a single environment and combined environment GWAS with values above the exploratory threshold (Supplementary Table S3). For TSW, 25 MTAs were identified, spanning all chromosomes except chromosome 10 (Chr A10). Nine, eight and five SNPs were significant only for Öjebyn, Lönnstorp and the combined environment, respectively. One SNP marker (*CM020890.1_33244505*) was significant for Öjebyn and the combined environment while another one (*CM020894.1_4615438*) was significant for Lönnstorp and the combined environment. Whereas, one marker (*CM020895.1_12959514*) was significant for Öjebyn, Lönnstorp and the combined environment. The SNP marker *CM020895.1_12959514* (MAF = 0.46) on Chr A08 displayed a stable association with TSW in each environment as well as the combined environment with levels of significance above the Bonferroni threshold (Table 2; Figure 6A).

**TABLE 2:** List of some SNP markers significantly associated at above-Bonferroni threshold levels with TSW, DTF or PSHR at least in two of the three cases (Öjebyn site, Lönnstorp site and combined environment) together with their SNP position within the corresponding chromosomes and minor allele frequencies (MAF).

Trait	Location	SNP	Chr	Pos (MB)	-log10(P values)	MAF
TSW	Lönnstorp	CM020895.1_12959514	8	12.96	16.57	0.46
	Öjebyn				12.04	
	Combined				9.36	
	Öjebyn	CM020890.1_33244505	3	33.24	9.01	0.12
	Combined				5.69	
	Combined	CM020894.1_4615438	7	4.62	8.88	0.05
	Lönnstorp				8.17	
DTF	Combined	CM020896.1_28000790	9	28.00	12.63	0.06
	Öjebyn				10.01	
	Öjebyn	CM020897.1_2008052	10	2.01	6.88	0.11
	Combined				6.70	
	Öjebyn	CM020897.1_2008052	10	2.01	6.88	0.11
	Lönnstorp				6.52	
PSHR	Combined	CM020888.1_28035981	1	28.04	9.30	0.10
	Lönnstorp				5.08	
	Combined	CM020890.1_26416338	3	26.42	5.39	0.19
	Lönnstorp				5.13	

TSW, thousand seed weight; DTF, days to flowering; PSHR, pod shattering resistance.

Accessions homozygous for the reference allele (A) at this locus had significantly higher TSW compared to accessions homozygous for the alternative allele (G) (*p < 0.01*) for the two sites as well as the combined environment (Figure 6B). The two SNP markers *CM020888.1_2486460* (MAF = 0.08) and *CM020888.1_2486421* (0.07) on Chr A01 with the same physical position (2.49 Mbp; only 39 bp apart) were significant for TSW for Öjebyn and Lönnstorp, respectively, and hence they may refer to the same minor QTL. The two SNP markers on Chr A07, CM020894.1*_4615438* (MAF = 0.05) and *CM020894.1_11913677* (MAF = 0.11) were also significantly associated with TSW (Supplementary Table S3; Figure 6A). CM020894.1*_4615438* may be considered stable, as it was significant for Lönnstorp and the combined environment.

**FIGURE 7:**
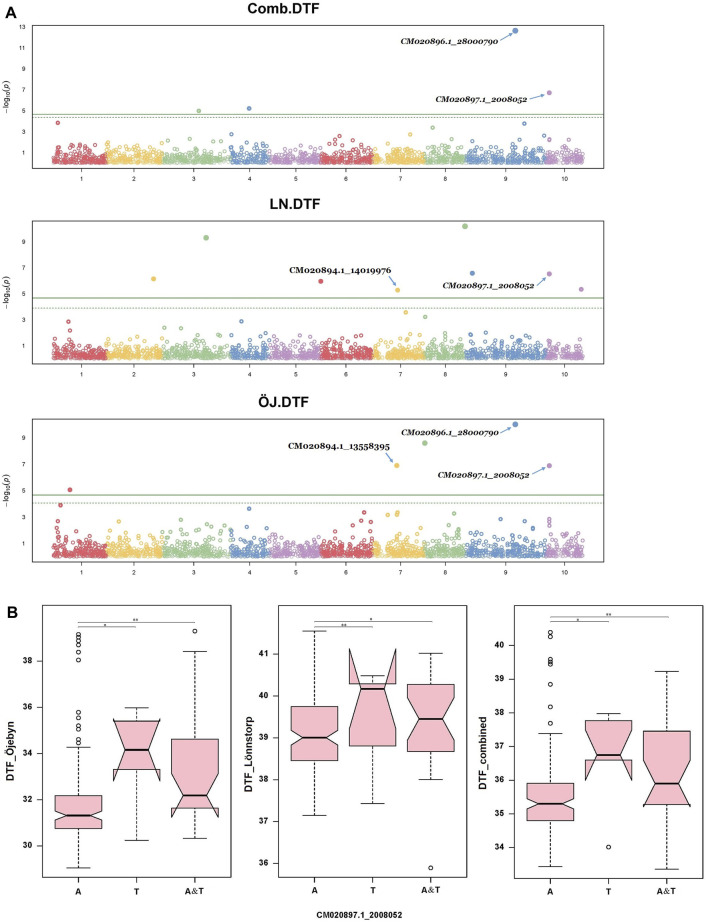
Manhattan plot **(A)** depicting significant SNP markers across the ten chromosomes associated with days to flowering (DTF) identified for the Öjebyn site, Lönnstorp site and combined environment, and box plots **(B)** depicting significant variation in TSW between groups of turnip rape accessions grouped based on whether they have only allele A or allele T or both at the SNP marker *CM020895.1_12959514*, for the two sites and combined environment. *p < 0.05; **p < 0.01.

DTF is a crucial trait for improvement in turnip rape, particularly in northern temperate regions via shortening days to flowering and maturity. Seventeen MTAs were identified for this trait for the two single environments and the combined environment involving 14 SNP markers across all chromosomes except Chr A05. The SNP marker *CM020896.1_28000790* (MAF = 0.06) on chromosome A09 was significant for Öjebyn and the combined environment with −*logP* values of 12.6 and 10, respectively (Supplementary Table S3; Figure 7A). The two closely located markers on Chr A07, *CM020894.1_13558395* (13.6 Mbp) and *CM020894.1_14019976* (14 MbP) were significantly associated with DTF for Öjebyn and Lönnstorp, respectively (Supplementary Table S3; Figure 7A). The SNP marker *CM020897.1_2008052* (MAF = 0.11) on Chr A10 was significant for Öjebyn, Lönnstorp and the combined environment, and hence is a stable marker for DTF. Analysis of variance revealed that accessions homozygous for allele A flowered earlier than accessions homozygous for allele T for both sites and the combined environment (*p < 0.01*) (Figure 7B).

Silique-related traits, pod resistance (PSHR), silique length (SL), seeds per silique (SS) and number of siliques (NS) were also evaluated for MTA, and several MTAs were identified except for NS. For PSHR, SL and SS, 16, 7 and 3 significant SNP markers, respectively, were identified across the two sites and the combined environment (Supplementary Table S3). SNP markers significantly associated with PSHR were distributed across all chromosomes except Chr A07. The SNP markers *CM020888.1_28035981* and *CM020890.1_26416338* on chromosomes 1 and 3, respectively, were significantly associated with PSHR for the Lönnstorp site and combined environment.

Other traits evaluated in the current study were yield (YLD), plant height (PH) and days to maturity (DTM). Yield and days to maturity were evaluated only for the Lönnstorp site. Five MTAs were identified for YLD on chromosomes 4, 5, 7, and 9, while 13 MTAs were identified for PH with associated SNPs distributed across all chromosomes except Chrs A04 and A07. For DTM, three MTAs were detected on Chr A02, A04 and A07 (Supplementary Table S3).

**FIGURE 8:**
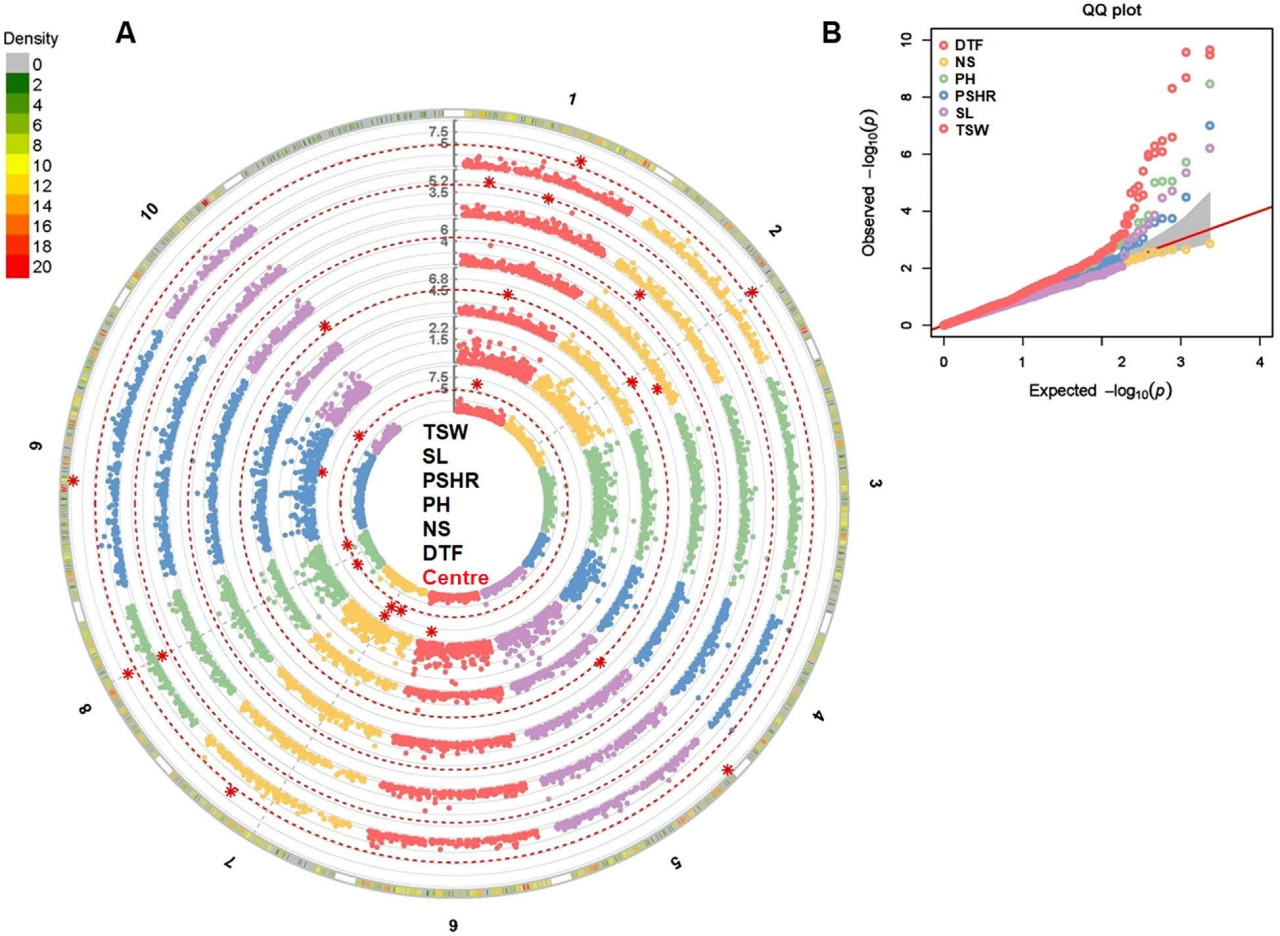
Phenotypic plasticity-based GWAS analysis for six agronomic traits evaluated across the two locations. Manhattan plot **(A)** with major marker-trait associations and Q-Q plots **(B)** of the studied traits. DTF, days to flowering; NS, number of siliques; PH, plant height; PSHR, pod shattering resistance; SL, silique length; TSW, thousand seed weight.

The GWAS analysis was performed using the phenotypic plasticity values estimated for traits evaluated at two locations (Figure 8). This analysis identified 23 major marker-trait associations (MTAs) for five traits (Supplementary Table S4). Given the limited number of tested environments (two), the majority of these MTAs (15) were also detected on the previous phenotypic BLUP-based GWAS analysis.

### Accuracy of the genomic estimated breeding values

**FIGURE 9:**
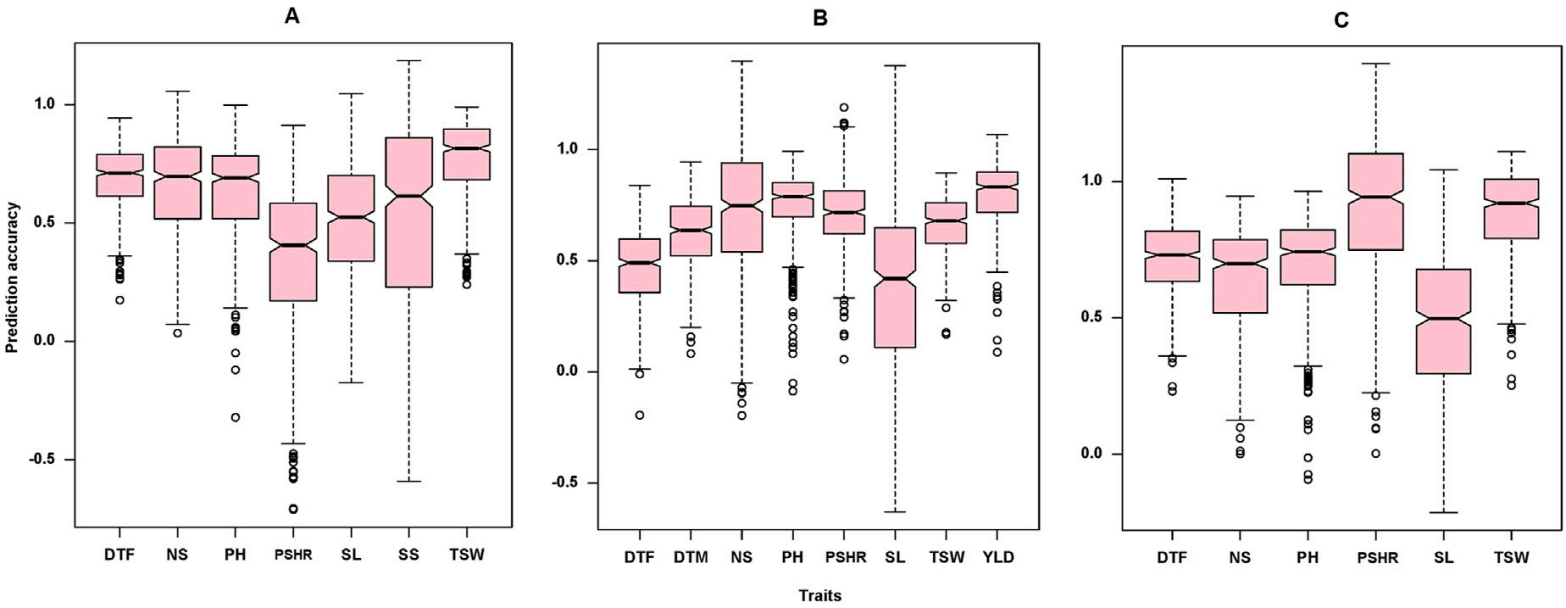
Box plots showing the distribution of genomic prediction accuracy estimated for the nine traits studied for the Öjebyn site **(A)**, Lönnstorp site **(B)**, and the combined environment **(C)**. DTF, days to flowering; NS, number of siliques; PH, plant height; PSHR, pod shattering resistance; SL, silique length; SS, number of seeds per silique; TSW, thousand seed weight; DTM, days to maturity; YLD, yield per plot.

The GWAS analysis conducted in this study successfully identified marker-trait associations for eight out of the nine traits examined, demonstrating the effectiveness of the marker set employed. Consequently, we tested the effectiveness of these marker set for genomic prediction of the target traits in order to promote the integration of genomic selection into turnip rape breeding programs. The GWAS analysis did not detect significant MTAs for the number of siliques (NS) at the significant threshold applied. Interestingly, the genomic-based prediction accuracy for this trait was 0.67, 0.73 and 0.64 for Öjebyn, Lönnstorp and the combined environment, respectively (Table 3). In general, the genomic prediction accuracy for the studied traits was promising (Table 3; Figure 9). For instance, the prediction accuracy for DTF for Öjebyn, Lönnstorp and the combined environment was 0.71, 0.47 and 0.71, respectively. For TSW, it was 0.77, 0.65 and 0.88 for Öjebyn, Lönnstorp and the combined environment, respectively. Moderate to low prediction accuracy was recorded for silique length, 0.52 (Öjebyn site), 0.37 (Lönnstorp site) and 0.48 (combined environment). On the other hand, the accuracy of genomic prediction for pod-shattering (PSHR) ranged from low to high (0.35–0.91) across the two sites and combined environment. Seed yield was evaluated only for the Lönnstorp site, and the genomic prediction accuracy for this trait was interestingly high (0.8) (Table 3).

**TABLE 3:** Average predictive ability and prediction accuracy of the model used for the analysis of nine phenotypic traits for the two sites (Öjebyn and Lönnstorp) separately and for the combined environment.

Location	Traits	Average predictive ability	Average prediction accuracy
Öjebyn	DTF	0.69	0.71
	NS	0.51	0.67
	PH	0.51	0.63
	PSHR	0.29	0.35
	SL	0.37	0.52
	SS	0.36	0.53
	TSW	0.75	0.77
Lönnstorp	DTF	0.43	0.47
	DTM	0.55	0.63
	NS	0.34	0.73
	PH	0.7	0.75
	PSHR	0.44	0.71
	SL	0.17	0.37
	TSW	0.59	0.65
	YLD	0.67	0.8
Two locations combined	DTF	0.64	0.71
	NS	0.29	0.64
	PH	0.65	0.7
	PSHR	0.52	0.91
	SL	0.36	0.48
	TSW	0.75	0.88

DTF, days to flowering; NS, number of siliques; PH, plant height; PSHR, pod shattering resistance; SL, silique length; SS, number of seeds per silique; TSW, thousand seed weight; DTM, days to maturity; YLD, yield per plot.

## Discussion

Turnip rape was among the largely cultivated edible and industrial oilseed crops globally until the 1970s. However, the crop is currently cultivated in very limited areas in India, Canada and Northern Europe (Kaur et al., 2021b). This is mainly because of the lack of breeding programs to develop cultivars with high yield and resistance/tolerance to recurring biotic and abiotic stresses. The current study was conducted at two locations in Sweden to evaluate the performance of turnip rape accessions for nine phenotypic traits commonly targeted in breeding. The panel comprised accessions of worldwide origin with diverse genetic backgrounds, including landraces, cultivars, breeding populations and wild populations. The single site-based and combined environment-based analysis of variance revealed highly significant variation among accessions for the majority of studied traits, and hence are suitable for marker-trait association (MTA) analysis via GWAS (Brachi et al., 2011). This facilitates the development of genomic tools that assist turnip rape breeding in order to accelerate genetic gains in target traits. Crop yield, nutritional quality and stress tolerance/resistance are quantitative traits regulated by several genes located in genomic regions referred to as quantitative trait loci (QTLs) (Consortium et al., 2003). These genes/QTLs further interact with each other and the environments where plants are grown, making phenotypic evaluation challenging. Multi-environment trial evaluation of genetically diverse accessions across different environments allows us to understand genotype stability and performance in these target traits. The current study was designed to assess the phenotypic and genetic variation of turnip rape accessions followed by GWAS and genomic prediction analyses.

The availability of whole genome assemblies of *B. rapa* (http://brassicadb.cn) has made it possible to discover genes/QTLs linked to economically valuable traits (Cai et al., 2017; Zhang et al., 2018). The current study has discovered several markers linked to the studied traits. Flowering and maturity time are among the most important traits in crop plants affecting biomass and seed yield as well as adaptation to different growing environments (Weis and Kossler, 2004; Gaudinier and Blackman, 2020). A large variation in flowering and maturity times has been observed among *B. rapa* germplasm leading to winter, semi-winter, and spring growth habits (Kaur et al., 2021b). Days to flowering (DTF) is a quantitatively inherited trait controlled by several genes with minor to major effects. Several of these genes have been identified and characterized in Brassicas, such as *FLOWERING LOCUS T (FT), FRUITFUL (FUL)*, *FLOWERING LOCUS C (FLC)*, *CONSTANS (CO)*, *FRIGIDA (FRI)* and *PHYTOCHROME (PHY)* (Long et al., 2007; Raman et al., 2013; Xiao et al., 2013; Raman et al., 2016; Gao et al., 2017). Previous studies showed that several genes (including those belonging to the same gene families) in Brassica and *Arabidopsis* control flower initiation and flowering (Schiessl et al., 2017). Studies on *B. rapa* showed that these genes are located on Chrs A01, A02, A03, A07 and A10 with a different number of copies (Schranz et al., 2002; Zhang et al., 2015; Leijten et al., 2018; Li et al., 2018). Similarly, the current GWAS study identified SNP markers significantly associated with DTF on Chrs A01 (single environment), A02 (single environment), A03 (combined environment), A07 (single environment) and A10 (both single and combined environments, and hence stable) (Supplementary Table S3). Li et al. (2018) reported 1,064, 510, and 524 putative orthologs of *Arabidopsis thaliana* flowering-time genes from *B. napus*, *B. rapa*, and *Brassica oleracea*, respectively, including several genes on Chr A09. Likewise, the present study identified the most significant marker-trait association (MTA) for DTF on the same chromosome. Day to maturity (DTM) was only analyzed for the Lönnstorp site and MTAs were identified on Chrs A04, A07 and A02. Similarly, Kaur et al. reported significant marker for this trait on Chr 02 (Kaur et al., 2021b). Zhou et al. (2017) also reported significant markers on Chrs A02, A03, A06, A07 and A09 (corresponding to the *B. rapa* genome) in rapeseed (*B. napus*) using a divers GWAS panel containing 300 inbred lines.

The GWAS studies carried out thus far in Brassicas for yield and related traits focused on *B. napus*. Therefore, the use of a different reference genome complicates the comparison of precise chromosome positions of identified QTLs with our study. In this study, 25 MTAs were detected for thousand seed weight (TSW) distributed across all *B. rapa* chromosomes, except Chr A10. Several previous researchers have identified QTLs for TSW on the A genome of *B. rapa* in line with the present study. In this study, Chrs A07, A08 and A09 contain highly significant QTLs for this trait, in line with previous studies (Yang et al., 2012; Li et al., 2014; Lu et al., 2017; Khan et al., 2019; Shen et al., 2019; Shi et al., 2019). The current study unveiled that all chromosomes except Chrs A04 and A07 carry significant SNP markers associated with PH. Similarly, Li et al. (2016) reported QTLs located on Chrs A03 and A05, while Zheng et al. (2017) reported QTLs on Chrs A02, A08, and A09, in their studies comprising 472 and 333 rapeseed accessions, respectively. Kaur et al. (2021b) reported PH MTAs on Chrs A01, A03, 05, A06, A08, A09 and 10 based on a panel of 195 inbred lines, representing natural and derived forms of *B*. *rapa* evaluated at two locations.

Regarding silique-related traits, our research focused on exploring silique length (SL), silique number (NS), number of seeds per silique (SS) and pod shattering (PSHR), which are major traits to improve in turnip rape breeding to enhance seed yield. Majority of previous studies on these traits are on *B. napus*, among Brassica crops, and hence we could only make chromosome-level comparisons. The GWAS analysis identified 16, 7 and 3 MTAs for PSHR, SL and SS, respectively, but none for NS. The MTAs for PSHR were identified on all chromosomes except on Chr A07. Similarly, Raman et al. (2014) identified PSHR related QTLs on Chrs A03, A07, and A09 in a study on 126 double haploid lines of *B. napus*. In agreement with our finding, several previous researches also reported MTAs/QTLs for PSHR on Brassica A genome chromosomes (Liu J. et al., 2016; Kaur et al., 2020; Raman et al., 2023). The current study identified SNP markers significantly associated with SL on Chrs A01, A03, A06, A08 and A09. Similarly, Dong et al. (2018) identified several markers associated with SL on Chrs A03, A08 and A09 from a GWAS study using 157 rapeseed inbred lines. In this study, a SNP marker with significant association with SL (combined environment) was found on Chr A09. This chromosome was recognized as a source of major QTLs for SL in several previous studies (Yang et al., 2012; Fu et al., 2015; Shi et al., 2019; Wang et al., 2019; Wang et al., 2021). We identified three MTAs for SS (Öjebyn site) on Chr A05 (two) and Chr A10. For YLD, five significant markers were identified (Lönnstorp site) on Chrs A04, A05, A07 and A09 (two). Previous studies have similarly identified SS related QTL/MTA on chromosomes 5 and 10 (Ding et al., 2012; Yang et al., 2016; Xin et al., 2021). In agreement with our study, a study on 124 recombinant inbred lines of *B. napus* revealed YLD-related QTLs on Chrs A04 and A05 (Ding et al., 2012).

Genomic prediction stands out as a valuable tool for accelerating genetic gains in crop breeding efforts (Alemu et al., 2024). This method has emerged as a preferred approach for polygenic quantitatively inherited traits, especially considering the challenges in breeding for such traits (Alemu et al., 2024). The current study explored the potential of genomic prediction in turnip rape breeding through a cross-validation study. The abundance of significantly associated SNP markers identified for the majority of studied traits in this study poses a challenge to their immediate application as selection and breeding tools in turnip rape. This observation prompted us to explore the potential of genomic prediction, which involves estimating the overall merit of individual accessions for improvement of a particular trait, rather than solely identifying specific MTAs. We applied an 80%–20% training-test set cross-validation approach to evaluate the accuracy of genomic prediction. The analysis provided promising results, with high prediction accuracy for the majority of targeted traits. No significant MTAs were detected via GWAS for the number of siliques per plant. However, higher genomic prediction accuracy was recorded for this trait, with values of 0.67, 0.73, and 0.63 for Öjebyn, Lönnstorp, and the combined environments, respectively. Similar to the GWAS studies, the majority of previous genomic prediction studies on Brassicas focused on estimating the GEBVs of hybrid *B*. *napus* (Würschum et al., 2014; Wei et al., 2016; Yang et al., 2016; Szwarc et al., 2023). Thus, the present study provides an interesting insight into the potential applications of genomic prediction in the breeding of turnip rape.

In summary, our study investigated the utilization of genomic tools to enhance turnip rape breeding. We evaluated a panel of 170 accessions representing a global turnip rape gene pool via field trials at two distinct locations, followed by the identification of MTAs and GEBVs through GWAS and genomic prediction studies, respectively. A total of 65 SNP markers were identified as significantly associated with eight of the nine traits studied. Notably, the genomic prediction accuracy for most targeted traits was high, suggesting the potential of genomics-driven breeding in expediting genetic gains in turnip rape.

## Data Availability

The datasets presented in this study can be found in online repositories. The names of the repository/repositories and accession number(s) can be found in the article/Supplementary material. The SNP markers are deposited at the following link: https://figshare.com/articles/dataset/Turnip_rape_SNP_marker_MAF0_05Hetrozygosity20_/25847716.
